# A rare case report of pigment gallstones and a concomitant gastric gastrointestinal stromal tumour

**DOI:** 10.1093/jscr/rjab637

**Published:** 2022-02-23

**Authors:** Yagan Pillay

**Affiliations:** Department of Surgery, University of Saskatchewan, Saskatoon, Saskatchewan, Canada

## Abstract

Gastric gastrointestinal stromal tumours (GISTs) with cholelithiasis in the English literature are quite rare with a few published case reports. Their concurrent surgical management has also been debated as the clinical symptoms are often ascribed to one of the pathologies. All case reports to date have reported on cholesterol cholelithiasis. We would like to present a first case report of pigment cholelithiasis and a gastric GIST and their concurrent laparoscopically management. This was facilitated due to the patient’s request to deal with both pathologies as well as the nebular signs concerning the symptoms that she presented with. We were able to undertake the dual surgeries without compromising her oncological outcomes.

## INTRODUCTION

Gastrointestinal stromal tumours (GISTs) are the commonest mesenchymal tumours of the gastrointestinal tract. With a recorded incidence of 0.1–3% of all gastrointestinal malignancies they remain quite rare [1]. They arise from the interstitial cells of Cajal and the majority are located in the stomach [1]. Seventy percent of gastric GISTs arise in the gastric body as in this case report [1]. Pigmented gallstones comprise between 2 and 30% of all gallstones and their aetiology involves excessive bilirubin destruction [2]. We present the following case in accordance with the CARE reporting checklist.

## CASE REPORT

A 64-year-old female patient was referred for surgical consultation of her chronic abdominal pain. The pain originated in her epigastrium and radiated to the back. There was no food pain association and the nature of the pain was described as spasmodic.

The pain was intermittent in nature and had been present for 1 year punctuated by the occasional emergency room visit for intravenous analgesic control. She had symptoms of dyspepsia and early post prandial satiety but no acid reflux or dysphagia.

**Figure 1:**
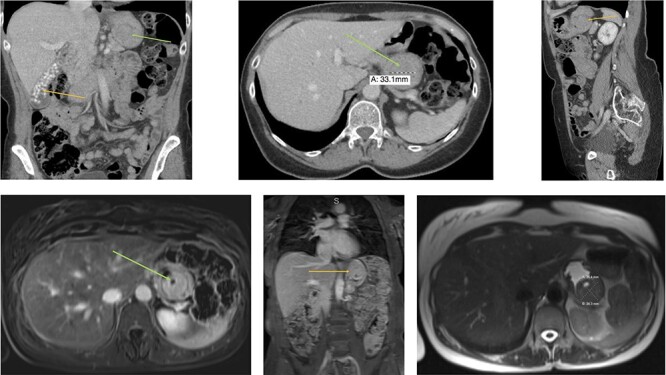
Radiology (**a**) CT scan coronal view showing the gallbladder filled with radiolucent stones (orange arrow) and the GIST in the stomach (green arrow). (**b**) CT scan axial view showing the gastric GIST in the submucosal tissue (green arrow). (**c**) CT scan sagittal view showing the gastric GIST on the posterior wall of the stomach (orange arrow). (**d**) MRI axial view showing the tumour with a pneumatocele (green arrow). (**e**) MRI coronal view showing the tumour on the posterior wall of the stomach (orange arrow) with the visible pneumatocele. (**f**) T2 weighted MRI axial view showing the GIST dimensions.

**Figure 2:**
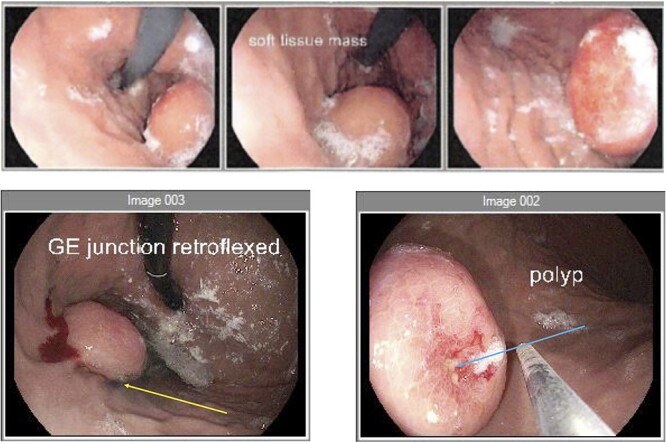
Endoscopy (**a**) gastroscope showing the GIST on the posterior gastric wall and its relationship to the gastro-oesophageal junction. (**b**) second gastroscope with injection of methylene blue (yellow arrow) to mark the tumour borders. (**c**) mucosal ulceration due to tumour enlargement (blue arrow) on the second gastroscope 6 weeks later.

An abdominal examination revealed no palpable lesions or abdominal wall hernia. She had no clinical signs of an acute abdomen. Her medical history was significant for asthma, dermatomycosis and endometriosis. Radiological imaging, with an abdominal ultrasound confirmed cholelithiasis but no cholecystitis or choledocholithiasis. The ultrasound also showed an incidental gastric lesion in the posterior wall of the stomach. A subsequent computerized tomography (CT) scan showed an isolated lesion in the posterior wall of the stomach and no obvious gastric lymphadenopathy (Fig. 1a–c). Radiolucent gallstones were also visualized on the CT scan (Fig. 1a). Differential diagnosis of the gastric lesion included a leiomyoma or a GIST. Upon discussion with an interventional radiologist, the gastric lesion was deemed radiologically inaccessible to percutaneous biopsy. A magnetic resonance imaging (MRI) scan was performed to confirm the diagnosis. MRI showed a well-circumscribed soft tissue lesion in the posterior wall of the stomach in keeping with a gastric GIST (Fig. 1d–f).

**Figure 3:**
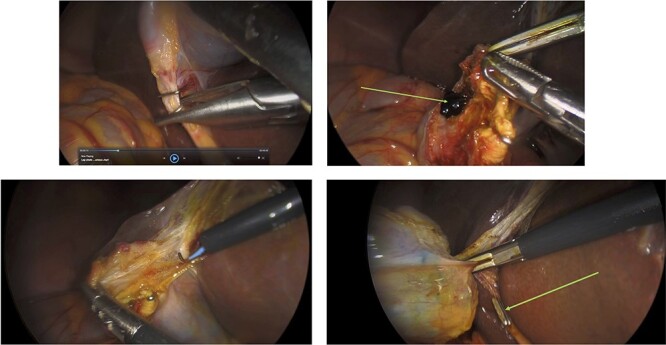
Surgery: laparoscopic cholecystectomy. (**a**) clipping of the common bile duct. (**b**) retrograde dissection of the fundus of the gallbladder. (**c**) iatrogenic gallbladder injury and spillage of pigment stones (green arrow). (**d**) completion of the retrograde dissection with visualization of the cystic duct stump (green arrow).

**Figure 4:**
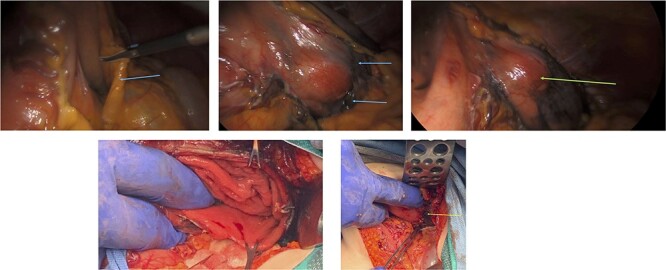
Surgery: gastric wedge resection. (**a**) laparoscopic entry into the lesser sac through the gastro-colonic ligament (blue arrow). (**b**) methylene blue markings visible on the serosal layer of the stomach (blue arrows) to delineate the tumour margins. (**c**) complete mobilization of the gastro-colic and gastro-splenic ligament and visualization of the gastric GIST (green arrow) on the posterior wall of the stomach. (**d**) macroscopically clear tumour margins post GIST resection. (**e**) posterior gastric wall sutured closure (yellow arrow).

**Figure 5:**
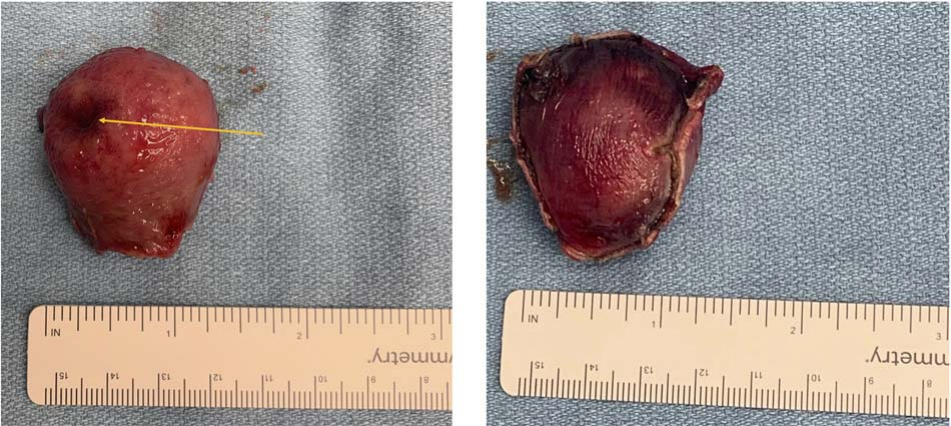
Gross pathology. (**a**) mucosal view with ulceration of the gastric mucosa (orange arrow). (**b**) serosal view showing the gastric serosal layer.

An elective gastroscope showed a soft tissue mass on the posterior wall of the body of the stomach (Fig. 2a). This appeared to be an underlying submucosal tumour with no mucosal involvement. Biopsies taken showed a normal mucosa with no malignant or premalignant mucosal pathology. A complete pathological diagnosis could not be ascertained and after an extensive discussion with the patient and her family, conservative management was contemplated rather than a surgical resection. Her symptom profile did not fit clearly with either biliary colic or a gastric GIST. Three months later the patient returned to consider surgery as the pain was not subsiding. She had a second gastroscope to mark the tumour margins with methylene blue as part of her workup for a laparoscopic gastric wedge resection (Fig. 2b). The mucosa over the lesion now showed an ulceration which we ascribed to excessive tumour growth (Fig. 2c). She also requested a concomitant laparoscopic cholecystectomy as we could not clearly elucidate the aetiology behind her abdominal pain. An informed consent was signed along with permission to record the surgical procedure. The laparoscopic cholecystectomy, initially performed, was uneventful. This was a combined antero-retrograde cholecystectomy (Fig. 3a–d). The greater curvature of her stomach was mobilized laparoscopically until the lesion was clearly visualized on the posterior wall with the aid of the methylene blue dye injected at endoscopy (Fig. 4a–c). The decision was then made to convert to open surgery and the lesion excised with a 1 cm margin (Fig. 4d). The gastrotomy wound was closed in two layers with a 2–0 polydioxanone absorbable running suture (Fig. 4e). Her physiological recovery was unremarkable, and she was discharged home on post-operative Day 5. Pathology confirmed the presence of a gastric GIST with clear surgical margins. Gallbladder pathology showed a chronic cholecystitis and pigment stones (Fig. 5).

## DISCUSSION

Gastric GISTs and cholelithiasis as a clinical presentation are quite sparse in the English literature with a few published case reports [3]. All reports have involved cholesterol stones and to our knowledge, this is the first case report of pigment stones with a concomitant GIST. The aetiology of the pigment stones have not been elucidated. Pigment stones arise as a result of the excessively high levels of bilirubin delivered to the liver. This is due to haemoglobin breakdown from diseases such as sickle cell disease and thalassemia [4]. This remains an incidental finding at radiology and to date no correlation between cholelithiasis and gastric GISTs have been demonstrated.

There has been an increase in the incidence of gastric GISTs and a Surveillance, Epidemiology and End Results (SEER) database study showed an increase from 0.55 to 0.78/100 000 population between 2001 and 2011 [5]. A German study on consecutive autopsies showed an incidence of 22.5% in individuals over age 50 for GISTs <1 cm in size [6].

They are usually identified incidentally through radiological imaging, endoscopic intervention or during surgery. They can also be identified as a complication of excessive growth through tumour bleeding or perforation. Their management, if asymptomatic, depends on their risk stratification which includes their mitotic index, tumour size and location [7]. A clear surgical margin and intraoperative tumour rupture remains the best predictor of local recurrence [7].

Surgical resection of gastric GISTs usually involves a wedge resection of the stomach without nodal resection as tumour metastasis is through direct extension and rarely through the gastric lymph nodes [8]. Tumours of the lesser curvature and pylorus if surgically excised through a wedge resection can predispose to gastric stenosis and consideration should be given to a distal gastrectomy [8]. A laparoscopic resection should be undertaken in experienced hands and in some centres a laparoscopic approach is the standard of care irrespective of tumour size [9].

The decision to convert to open surgery was based on the medial margin of the tumour being quite close to the lesser curvature of the stomach. We could not ascertain, laparoscopically if we would cause a stenosis by involving the lesser curvature in our gastrotomy wound and its subsequent closure. Our patient has been referred to the surgical oncology clinic and started on a 3-year course of an oral tyrosine kinase inhibitor (Imatinib©). Her tumour’s mitotic index was five per high powered field, indicative of a high-risk tumour for metastasis and recurrence. The dual surgeries performed for two different pathologies did not adversely affect her oncological outcome as evidenced by the clear tumour margins and an uneventful recovery.

## CONCLUSIONS

We present a rare case of pigment cholelithiasis and a gastric GIST. These dual pathologies were laparoscopically managed, concurrently, with excellent oncological outcomes.

## FOOTNOTE

Reporting Checklist: The authors have completed the CARE reporting checklist.
